# Supplementary material for: Adam9-deficient retinal pigment epithelium pseudopods maintain photoreceptor outer segment renewal despite subretinal space expansion
Source: J Clin Invest. 2026 Feb 5;136(7):e196705. doi: 10.1172/JCI196705 (PMC13067945; doi:10.1172/JCI196705)
Supplement: Supplemental data [file jci-136-196705-s075.pdf]

Supplemental material

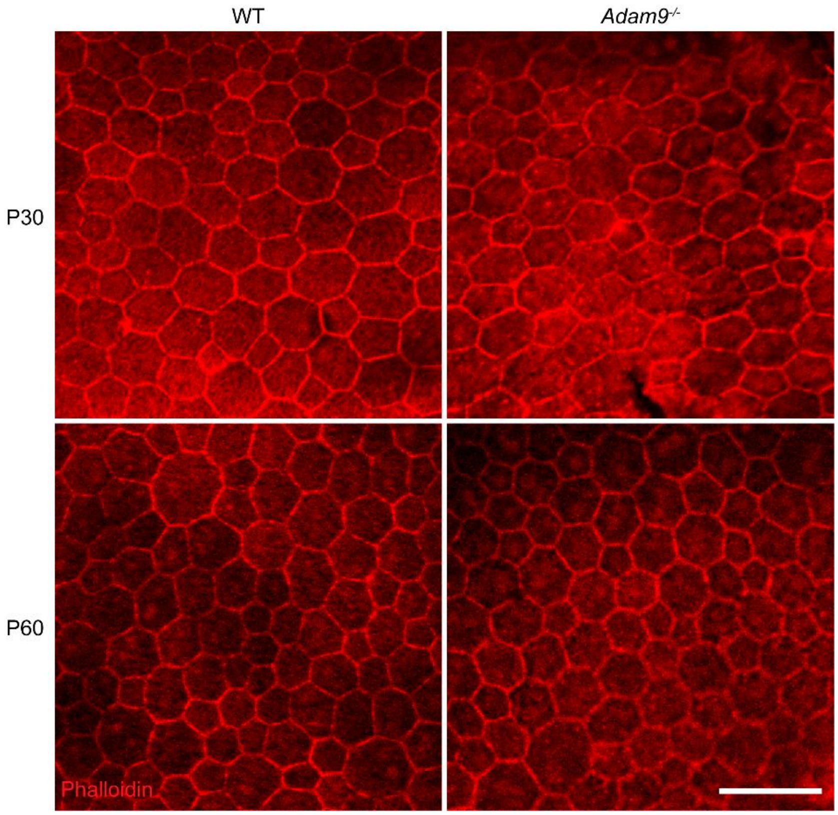

Supplemental Figure 1. Loss of ADAM9 does not lead to overt pathology in the RPE monolayer at early timepoints

Representative immunofluorescence images of RPE flatmounts stained with phalloidin to visualize RPE cell borders from WT and *Adam9*$^{-/-}$ mice at P30 and P60. For each genotype at each timepoint, three retinas from separate mice were analyzed. Scale bar: 50 μm.

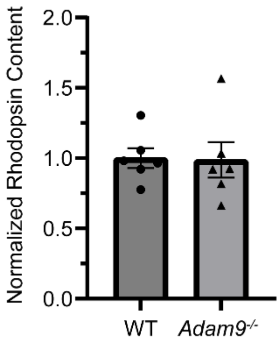

Supplemental Figure 2. Loss of ADAM9 does not affect the total rhodopsin content in the retina prior to photoreceptor degeneration

Total rhodopsin content of dissected eyecups was determined by difference spectroscopy and normalized to the average WT value. Unpaired t-test showed no statistically significant difference in rhodopsin content between WT and *Adam9*$^{-/-}$ eyecups at P30 ($p = 0.9321$). For each genotype, six eyecups were analyzed. Error bars represent mean ± SEM.

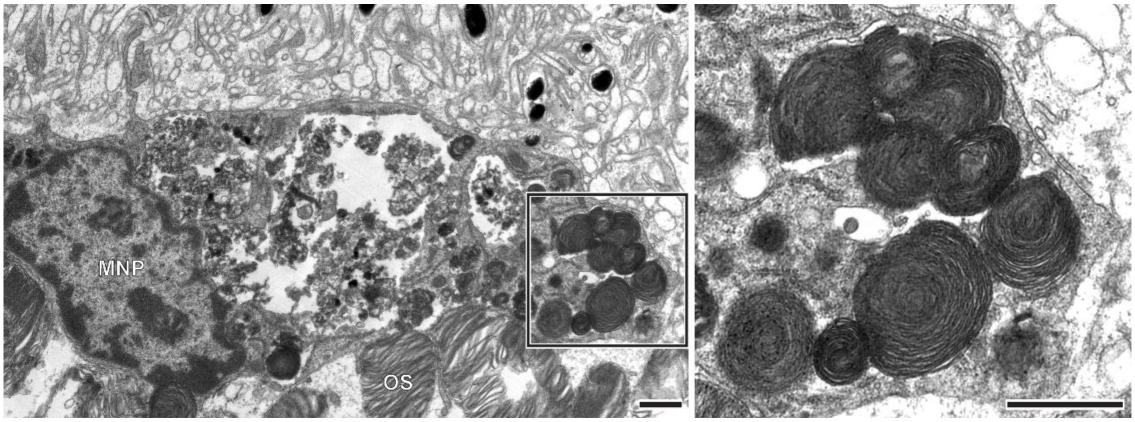

Supplemental Figure 3. Subretinal mononuclear phagocytes are capable of ingesting outer segment material

Representative TEM images of a mononuclear phagocyte (MNP) in the subretinal space of an *Adam9*$^{-/-}$ mouse at P180. Three retinas from separate mice were analyzed. Boxed area containing several phagosomes is shown at higher magnification to the right. OS: outer segment. Scale bars: 1 μm.

Movie 1. Reconstructed tomograms across four serial sections from an *Adam9*$^{-/-}$ retina – Example 1

Shown is an ~2 µm thick volume of the *Adam9*$^{-/-}$ retina with an isotropic resolution of 7.4 nm.

Movie 2. Reconstructed tomograms across four serial sections from an *Adam9*$^{-/-}$ retina – Example 2

Shown is an ~2 µm thick volume of the *Adam9*$^{-/-}$ retina with an isotropic resolution of 7.4 nm.
